# *Lactiplantibacillus plantarum* Reduced Renal Calcium Oxalate Stones by Regulating Arginine Metabolism in Gut Microbiota

**DOI:** 10.3389/fmicb.2021.743097

**Published:** 2021-09-24

**Authors:** Yu Liu, Xi Jin, Lei Tian, Zhongyu Jian, Yucheng Ma, Liang Cheng, Yaqian Cui, Hong Li, Qun Sun, Kunjie Wang

**Affiliations:** ^1^Laboratory of Reconstructive Urology, Department of Urology, Institute of Urology, West China Hospital, Sichuan University, Chengdu, China; ^2^Key Laboratory of Bio-Resources and Eco-Environment of Ministry of Education, College of Life Sciences, Sichuan University, Chengdu, China

**Keywords:** renal stones, calcium oxalate, *Lactiplantibacillus plantarum*, gut microbiota, arginine

## Abstract

Renal calcium oxalate (CaOx) stones are a common kidney disease. There are few methods for reducing the formation of these stones. However, the potential of probiotics for reducing renal stones has received increasing interest. We previously isolated a strain of *Lactiplantibacillus plantarum* N-1 from traditional cheese in China. This study aimed to investigate the effects of N-1 on renal CaOx crystal deposition. Thirty rats were randomly allocated to three groups: control group (ddH2O by gavage), model group [ddH2O by gavage and 1% ethylene glycol (EG) in drinking water], and *Lactiplantibacillus* group (N-1 by gavage and 1% EG in drinking water). After 4 weeks, compared with the model group, the group treated with N-1 exhibited significantly reduced renal crystals (*P* < 0.05). In the ileum and caecum, the relative abundances of *Lactobacillus* and *Eubacterium ventriosum* were higher in the control group, and those of *Ruminococcaceae UCG 007* and *Rikenellaceae RC9* were higher in the N-1-supplemented group. In contrast, the relative abundances of *Staphylococcus*, *Corynebacterium 1*, *Jeotgalicoccus*, *Psychrobacter*, and *Aerococcus* were higher in the model group. We also predicted that the arginase level would be higher in the ileal microbiota of the model group than in the N-1-supplemented group with PICRUSt2. The arginase activity was higher, while the level of arginine was lower in the ileal contents of the model group than in the N-1-supplemented group. The arginine level in the blood was also higher in the N-1-supplemented group than in the model group. *In vitro* studies showed that exposure to arginine could reduce CaOx crystal adhesion to renal epithelial HK-2 cells. Our findings highlighted the important role of N-1 in reducing renal CaOx crystals by regulating arginine metabolism in the gut microbiota. Probiotics containing *L. plantarum* N-1 may be potential therapies for preventing renal CaOx stones.

## Introduction

Renal stones are a common kidney disease worldwide that can cause pain, urinary tract infection, chronic renal disease, or even loss of renal function. The prevalence of these stones has been increasing, being approximately 6.4% in China (Zeng et al., 2017), 6.3% in the United States (Tundo et al., 2018), and 5% in Europe (Sorokin et al., 2017). Treatments for renal stones include drugs, extracorporeal shock wave lithotripsy, flexible ureteroscopic lithotripsy, and percutaneous nephrolithotomy. Although most renal stones can be removed successfully using these therapies, the recurrence rate is high at 53% after 5 years (Liu et al., 2018). The annual cost of renal stone disease in 2000 in the United States was approximately $2.81 billion and will continue to rise (Antonelli et al., 2014).

The main component of renal stones is calcium oxalate (CaOx), which accounts for approximately 80% of renal stones (Knoll et al., 2011). Only a few renal CaOx stones are caused by primary hyperoxaluria due to the lack of enzymes alanine–glyoxylate-aminotransferase or glyoxylate reductase/hydroxypyruvate reductase in the liver (Hoppe, 2012). Some renal CaOx stone patients are concurrently diagnosed with hyperparathyroidism, resulting in excessive parathyroid hormone secretion and hypercalciuria, followed by renal stones (Minisola et al., 2018). However, the reason for renal CaOx stones in the majority of patients is still unknown.

Oxalate and its precursors in food are absorbed into the blood, and then excreted into the urine (Sadaf et al., 2017). Recently, some studies have focused on the association between gut microbiota and renal CaOx stones. It was reported that *Oxalobacter formigenes* could degrade oxalate into formate through formyl-CoA transferase and oxalyl-CoA decarboxylase (Sadaf et al., 2017). The urinary oxalate level decreased in rats with hyperoxaluria after supplementation with *O. formigenes* (Sidhu et al., 2001; Hatch et al., 2011). However, the results of clinical trials were contrary to each other. A randomised phase II/III study reported that the changes in urinary oxalate levels were not different between primary hyperoxaluria patients treated with or without *O. formigenes* (Milliner et al., 2018). Another ongoing Phase II study found that 24 months *O. formigenes* administration substantially decreased plasma oxalate concentrations when compared with an untreated natural control cohort (Hoppe et al., 2021).

In addition, probiotics containing *Lactobacillus* and *Bifidobacterium* have demonstrated significant potential for treating various diseases. It was reported that the intake of probiotics could control oxidative stress and systemic inflammation in chronic kidney disease patients (Lopes et al., 2018). Some studies found that the administration of lactic acid bacteria reduced urinary oxalate levels in nephrolithiasis patients (Campieri et al., 2001; Lieske et al., 2005). However, several studies reported the different results that lactic acid bacteria neither reduced urinary oxalate excretion nor plasma oxalate concentration (Goldfarb et al., 2007; Siener et al., 2013). A probiotic supplement (VSL#3^®^), containing *Streptococcus thermophilus*, *Bifidobacterium*, and *Lactobacillus*, could reduce gastrointestinal oxalate absorption (Okombo and Liebman, 2010; Al-Wahsh et al., 2012). These evidences indicated that the supplementation with probiotics may be a potential strategy for preventing renal stones, which need further studies. We previously isolated seven strains of *Lactiplantibacillus plantarum* from two kinds of traditional cheeses in Daocheng, Sichuan, China (Liu et al., 2017), which were safe to use as probiotics. *L. plantarum K41* could inhibit the formation of *Streptococcus mutans* biofilms. The aim of the current study was to explore whether one of the strains, *L. plantarum* N-1, could reduce the formation of renal crystals and to study the role of gut microbiota during this process.

## Materials and Methods

### Culture of *Lactiplantibacillus plantarum* N-1

The *L. plantarum* N-1 strain preserved in glycerin was obtained from the Key Laboratory of Bioresources and Eco-environment of The Ministry of Education, Sichuan University. N-1 strains were cultured in 10 mL MRS medium (Difco, China) at an inoculation of 1% (v/v) for 18 h at 37°C. Then, the suspension of strains was centrifuged for 5 min at 3,000 × *g*. After two washes, the strains were resuspended in 10 mL saline solution. The bacterial suspension was adjusted to 10^8^ CFU/mL with a spectrophotometer and preserved in a centrifuge tube at 4°C for further use.

### Development of Rats With Renal Calcium Oxalate Stones and Administration of *Lactiplantibacillus plantarum* N-1

We purchased 6-week-old male Sprague–Dawley rats from Dossy Experimental Animals Co., Ltd. (Chengdu, Sichuan, China). The mean body weight of rats was 198 g. The rats were first acclimatised for 1 week before the experiment in the specific-pathogen-free facility at the Animal Experiment Center of West China Hospital, Sichuan University. The rats were given standard food and sterile water. We obtained ethical approval from the West China Hospital of Sichuan University Medical Research Ethics Committee (protocol numbers: 2017063A).

Thirty rats were randomly allocated to three groups: the control group, model group, and *Lactiplantibacillus* group. The control group rats were provided with 2 mL/kg ddH_2_O by gavage. Apart from ddH_2_O gavage, the rats in the model group had free access to drinking water with 1% (v/v) ethylene glycol (EG). In the *Lactiplantibacillus* group, the rats had drinking water with 1% EG and a suspension of N-1 (1 × 10^8^ CFU/mL, 2 mL/kg) by gavage once a day. After 4 weeks, 24 h urine of all the rats was collected using metabolic cages. Each urine sample was divided into three aliquots, which were supplemented with 0.5 mL hydrochloric acid (6 mol/L)/10 mL urine to prevent the degradation of oxalate (Wu et al., 2015). Then, we injected chloral hydrate [4% (w/v), 0.8 mL/100 g] intraperitoneally to euthanise the rats. Blood was drawn from the heart and centrifuged for 10 min (1,500 × *g*, 4°C) to obtain plasma. Renal tissues were fixed in 10% formaldehyde and embedded in paraffin. The remaining kidneys, plasma and intestinal contents in the ileum, caecum, and colon were stored at −80°C.

### Observation of Renal Crystals and Urinary Oxalate

Haematoxylin–eosin (HE) and von Kossa (VK) staining methods were used to observe the renal CaOx crystals. First, kidney sections (3–4 μm) were prepared and stained with HE following the manufacturer’s protocols for the staining kit (Thermo Fisher Scientific, United States). Second, VK staining was also performed to detect calcium crystals using a staining kit (Solarbio, Beijing, China). We analysed the staining images using a microscope (magnification, ×100; CARL ZEISS, Heidenheim, Germany). The HE staining scoring system reported by Xiang et al. (2015) and the VK staining scoring method using Image-Pro Plus 6 software were separately applied as semiquantitative and quantitative methods to evaluate renal crystals. Higher scores indicated more crystal deposition.

Urinary oxalate was measured using liquid chromatography–mass spectrometry. Briefly, 10 μL hydrochloric acid (12 mol/L) and 200 μL 1,2-diaminobenzene (5 mg/mL) were added to the 100 μL urine, which was then heated at 140°C for 40 min. When the sample was cooled to room temperature, it was added with 10 μL sodium hydroxide (10 mol/L) and centrifuged for 10 min (1,500 × *g*). Then, 100 μL supernatant was added with 300 μL acetonitrile and centrifuged for 10 min (1,500 × *g*). The oxalate was turned into 2,3-dihydroxyquinoxaline. The samples were examined using an LCMS-8040 system (Shimadzu, Kyoto, Japan).

### Examination of Gut Microbiota

The 16S rRNA gene sequencing technique was applied to analyse the gut microbiota. Total microbial DNA was extracted from faecal samples. Then, the V3–V4 regions of the 16S rRNA gene were amplified with polymerase chain reaction. The amplification products were sequenced on an Illumina MiSeq platform (Illumina, San Diego, CA, United States). The sequences were classified into different operational taxonomic units (OTUs) when their similarity was 97% or higher. The Silva 16S rRNA database (version 138) was used to identify the taxonomy of the OTUs.

### Determination of Amino Acids in the Ileal Contents and Blood

Amino acids were measured by liquid chromatography–mass spectrometry. We mixed 100 mg of ileal contents with 1 mL acetonitrile/methanol/water (2:2:1, v/v) and sonicated them for 30 min (4°C), followed by centrifugation for 20 min (15,000 × *g*, 4°C). Fifty microlitres of plasma and 200 μL of methanol acetonitrile/methanol (1:1, v/v) were mixed. We left the mixture to stand for 60 min before centrifugation for 20 min (15,000 × *g*, 4°C). Then, we applied the supernatant to UHPLC (Agilent, Santa Clara, CA, United States) and a QTRAP 5500 system (Sciex, Redwood, VA, United States). If the relative standard deviations of quality control (containing the same amount of contents from each sample) for all amino acids were less than 30%, the measurements were reproducible and stable. The information of amino acids standards used for liquid chromatography–mass spectrometry analysis was shown in Supplementary Table 1. The relative quantitative data for each amino acid was calculated based on the peak areas and retention times obtained from Multiquant software. The standards were used to calculate the retention time and identify amino acids.

### Measurement of Arginase Activity in the Ileal Contents

Arginase activity was determined using a colorimetric arginase activity assay kit (ab180877, Abcam, Cambridge, United Kingdom) according to the manufacturer’s protocol. Briefly, each sample well was added with 20 μL plasma, 20 μL assay buffer and 10 μL substrate mix. Each standard well was added with 50 μL prepared standards with different concentrations. The 96 well plate was incubated for 20 min at 37°C. Then each well was added with 50 μL reaction mix. Finally, the absorbance was measured on a microplate reader at OD = 570 nm in kinetic mode at 37°C. The arginase activity was calculated based on absorbance values and standard curve.

### Cell Culture and Calcium Oxalate Crystal Adhesion Assay

HK−2 cells were cultured in DMEM/F−12 supplemented with 10% foetal bovine serum at 37°C, 5% CO2 and saturated humidity. The cells were cultured in six-well culture plates and exposed to sodium oxalate (0.75 mM) and/or arginine (0.86, 1.72, 4.31, 8.61, 17.22, and 43.05 mM) for 24 h (Salama et al., 2019; Rattazzi et al., 2020). After that, the medium was changed to culture medium containing 40 μg/mL CaOx crystals for 10 min in a cell culture incubator. Then, the cells were washed with PBS thoroughly and examined under a bright field microscope (magnification, ×100; CARL ZEISS, Heidenheim, Germany). We selected five different fields to calculate the number of adhesive crystals.

### Statistics

Data are shown as the mean and standard deviation and were analysed by ANOVA and Student’s *t*-test. *P*-values < 0.05 were regarded as significantly different. GraphPad Prism (version 8) and R (version 3.6.1) were used for statistical analyses and to draw graphs.

## Results

### *Lactiplantibacillus plantarum* N-1 Reduced Renal Calcium Oxalate Crystals

**FIGURE 1:**
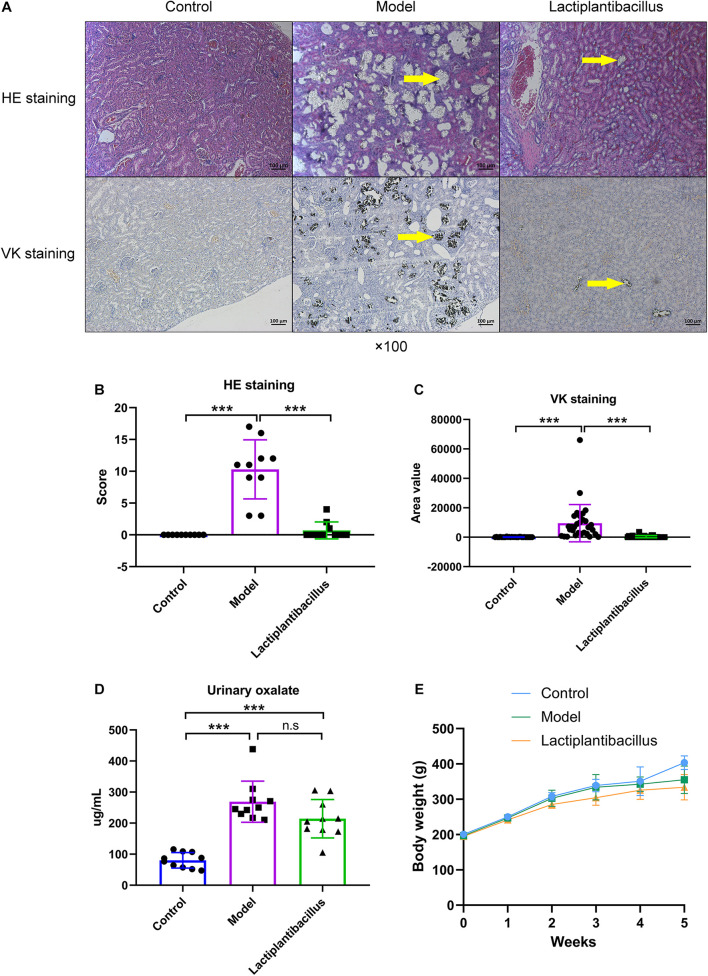
Haematoxylin–eosin (HE) staining and von Kossa (VK) staining of kidney tissue, urinary oxalate level and body weight of rats in the control group (*n* = 10), model group (*n* = 10), and *Lactiplantibacillus* group (*n* = 10). **(A)** HE and VK staining images of kidney tissues. **(B)** HE staining score and **(C)** VK staining area (three different sections of each staining were selected) of kidney tissues. **(D)** The urinary oxalate level in three groups. **(E)** The weight of the rats in the three groups per week from the beginning to the end of the experiments. Data are presented as the mean ± SD and were analysed using Student’s *t*-test between two groups. ***, *P* < 0.001; n.s, *P* > 0.05.

To explore the effect of N-1 on renal CaOx crystal formation, we provided model rats with N-1. After 4 weeks, HE staining showed that 1% EG successfully induced CaOx crystals in the kidney, which was also confirmed by VK staining. Supplementation with N-1 significantly reduced renal crystals (Figure 1A). The HE staining score and VK staining value (area) all increased significantly in the model group and decreased significantly in the N-1-supplemented group (*P* < 0.05) (Figures 1B,C). The level of urinary oxalate was higher in the model rats than in the controls (*P* < 0.05). However, the administration of N-1 did not affect urinary oxalate excretion (*P* > 0.05) (Figure 1D). The volume of 24 h urine was not statistically different between three groups (*P* > 0.05) (Supplementary Figure 1). The weights of all the rats increased steadily from the beginning to the end of the experiment. After 4 weeks, the weight of the model rats was lower than that of the control rats (*P* < 0.05), and they did not change after the administration of N-1 (*P* > 0.05) (Figure 1E).

### *Lactiplantibacillus plantarum* N-1 Reversed the Beta Diversity of the Gut Microbiota in the Ileum

To explore whether N-1 reduced renal crystals by regulating the gut microbiota, we next examined the gut microbiota. We found that the coverage indices were >0.99 in all groups at the OTU level, indicating that the sequencing depth was sufficient in all samples. The alpha diversity of the gut microbiota in the ileum, caecum, and colon was not different between the control, model, and N-1-supplemented groups (Supplementary Table 2).

**FIGURE 2:**
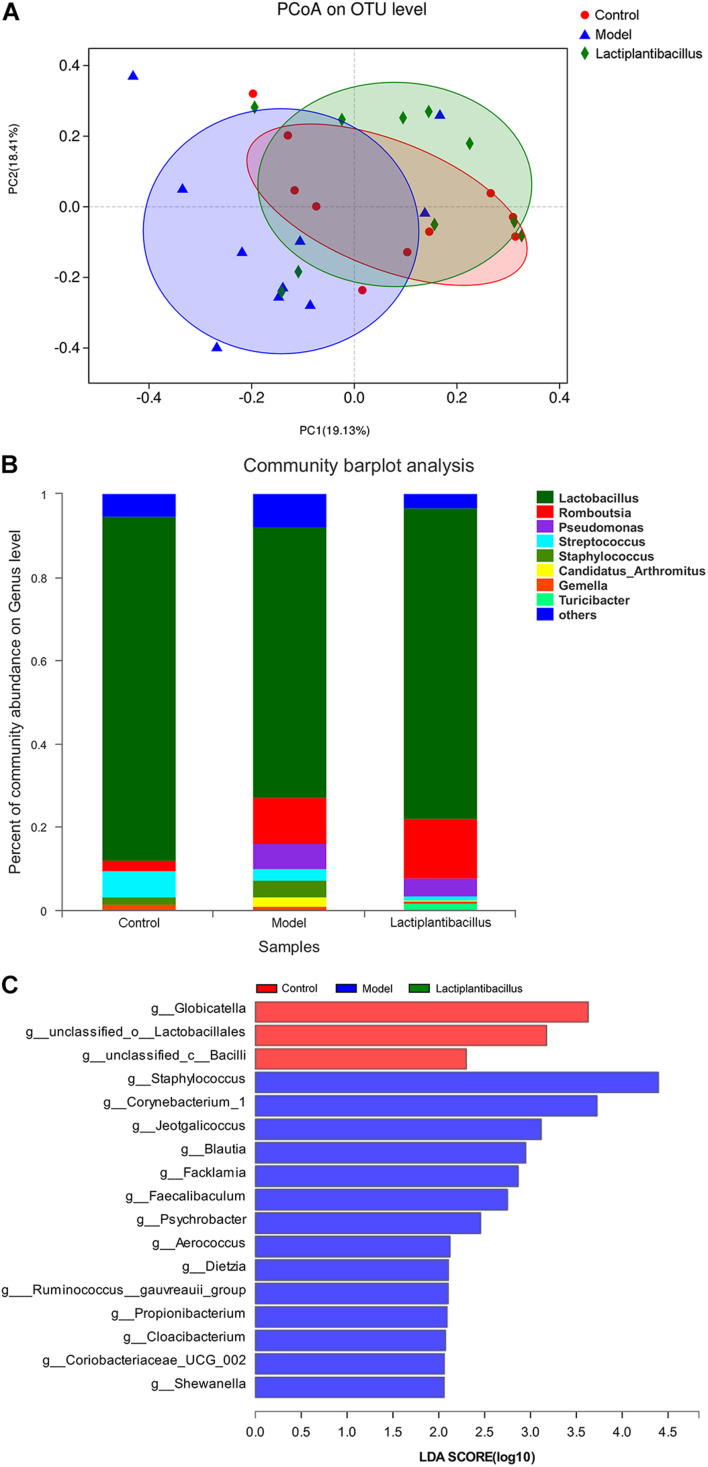
Analysis of the gut microbiota in the ileum between the control group (*n* = 10), model group (*n* = 10), and *Lactiplantibacillus* group (*n* = 10). **(A)** Beta diversity of the gut microbiota in the ileum between the three groups was assessed by principal coordinate analysis using the Bray–Curtis index. **(B)** The relative abundance of bacteria at the genus level in the ileum between the three groups is presented by the colourful columns. **(C)** The bacteria with higher relative abundance in the model group (red bars), model group (blue bar), and *Lactiplantibacillus* group (green bars) using linear discriminant analysis effect size (LEfSe) analysis at the genus level in the ileum.

The beta diversity of the gut microbiota was also evaluated by principal coordinate analysis (PCoA) using the Bray–Curtis index. In the ileum, PCoA showed significant separation between the control and model rats (*P* = 0.019, ADONIS). The beta diversity of the gut microbiota was different between the model group and the *Lactiplantibacillus* group (*P* = 0.045, ADONIS). The difference in the beta diversity between the control and N-1-supplemented groups decreased (*P* = 0.190, ADONIS) (Figure 2A). In the caecum and colon, although the beta diversity was also different between the control and model groups, it did not change in the N-1-supplemented group compared with the model group (*P* > 0.050, ADONIS) (Supplementary Figures 2A,B). This evidence indicated that N-1 could significantly affect the gut microbiota, especially in the ileum.

### *Lactiplantibacillus plantarum* N-1 Changed the Gut Microbiota

At the phylum level, Firmicutes was the main component (79–97%). At the genus level, *Lactobacillus* was the most common bacterium. The relative abundance of *Lactobacillus* decreased from 65–83% in the ileum to 16–36% in the caecum and to 18–46% in the colon. In the ileum, the percentage of *Lactobacillus* decreased from 83% in the control group to 65% in the model group and then increased to 75% in the N-1-supplemented group (Figure 2B). In the caecum and colon, its percentage decreased in the model and N-1-supplemented groups compared with the control group, without any difference between the model and N-1-supplemented groups (Supplementary Figures 2C,D). This evidence again suggested that N-1 significantly regulated the gut microbiota in the ileum but not in the caecum or colon.

At the genus level, we used linear discriminant analysis effect size (LEfSe) to explore the different bacteria between the control, model, and N-1-supplemented groups. In the ileum, *Staphylococcus*, *Corynebacterium 1*, *Jeotgalicoccus*, *Psychrobacter*, and *Aerococcus* were more abundant in the model group (Figure 2C). In contrast, the relative abundances of *Lactobacillus* and *Eubacterium ventriosum* were higher in the control group, and those of *Ruminococcaceae UCG 007* and *Rikenellaceae RC9* were higher in the N-1-supplemented group in the caecum (Supplementary Figure 3A) and colon (Supplementary Figure 3B).

### *Lactiplantibacillus plantarum* N-1 Regulated Metabolic Enzymes and Amino Acid Metabolism in the Gut

Apart from bacteria, we also used phylogenetic investigation of communities by reconstruction of unobserved states (PICRUSt2) to predict the metabolic enzymes of the gut microbiota and analysed their differences between the three groups. Using LEfSe, we found that the levels of 19 enzymes were higher in the ileum model group (such as arginase). In addition, the levels of 12 enzymes were increased in the ileum after the administration of N-1 (such as L-serine ammonia-lyase). However, there were fewer significantly different enzymes between the model and N-1-supplemented groups in the caecum and colon than in the ileum (Table 1).

**TABLE 1:** Higher predicted enzyme levels involved in the metabolic pathways of the gut microbiota in the ileum, caecum, and colon of the model and *Lactiplantibacillus* groups.

	Model		*Lactiplantibacillus*	
	EC number	Name	EC number	Name
Ileum	f.3.1.21.3	Type I site-specific deoxyribonuclease	f.4.3.1.17	L-Serine ammonia-lyase
	f.3.6.3.44	Xenobiotic-transporting ATPase	f.4.1.2.4	Deoxyribose-phosphate aldolase
	f.2.7.4.1	Polyphosphate kinase	f.3.5.1.2	Glutaminase
	f.1.6.99.3	NADH dehydrogenase	f.3.4.21.89	Signal peptidase I
	f.2.7.2.4	Aspartate kinase	f.4.4.1.16	Selenocysteine lyase
	f.5.3.1.6	Ribose-5-phosphate isomerase	f.5.1.3.2	UDP-glucose 4-epimerase
	f.2.7.7.9	UTP–glucose-1-phosphate uridylyltransferase	f.2.8.3.19	CoA:oxalate CoA-transferase
	f.2.4.2.52	Triphosphoribosyl-dephospho-CoA synthase	f.4.2.1.126	*N*-acetylmuramic acid 6-phosphate etherase
	f.4.1.1.20	Diaminopimelate decarboxylase	f.2.1.1.163	Demethylmenaquinone methyltransferase
	f.1.2.1.11	Aspartate-semialdehyde dehydrogenase	f.2.1.1.201	2-Methoxy-6-polyprenyl-1,4-benzoquinol methylase
	f.3.5.4.26	Diaminohydroxyphosphoribosylaminopyrimidine deaminase	f.3.4.21.102	C-terminal processing peptidase
	f.1.1.1.193	5-Amino-6-(5-phosphoribosylamino)uracil reductase	f.2.7.1.95	Kanamycin kinase
	f.2.3.1.81	Aminoglycoside *N*(3′)-acetyltransferase		
	f.4.1.3.42	(4S)-4-hydroxy-2-oxoglutarate aldolase		
	f.4.1.2.14	2-Dehydro-3-deoxy-phosphogluconate aldolase		
	f.5.1.1.7	Diaminopimelate epimerase		
	f.4.1.2.17	L-Fuculose-phosphate aldolase		
	f.2.7.1.45	2-Dehydro-3-deoxygluconokinase		
	f.3.5.3.1	Arginase		
Caecum	f.1.3.5.4	Fumarate reductase (quinol)	f.3.4.16.4	Serine-type D-Ala-D-Ala carboxypeptidase
			f.2.7.11.1	Non-specific serine/threonine protein kinase
			f.2.7.1.45	2-Dehydro-3-deoxygluconokinase
Colon	f.2.7.6.5	GTP diphosphokinase	f.2.7.7.13	Mannose-1-phosphate guanylyltransferase
	f.5.4.99.9	UDP-galactopyranose mutase		
	f.1.1.3.15	(S)-2-hydroxy-acid oxidase		

**FIGURE 3:**
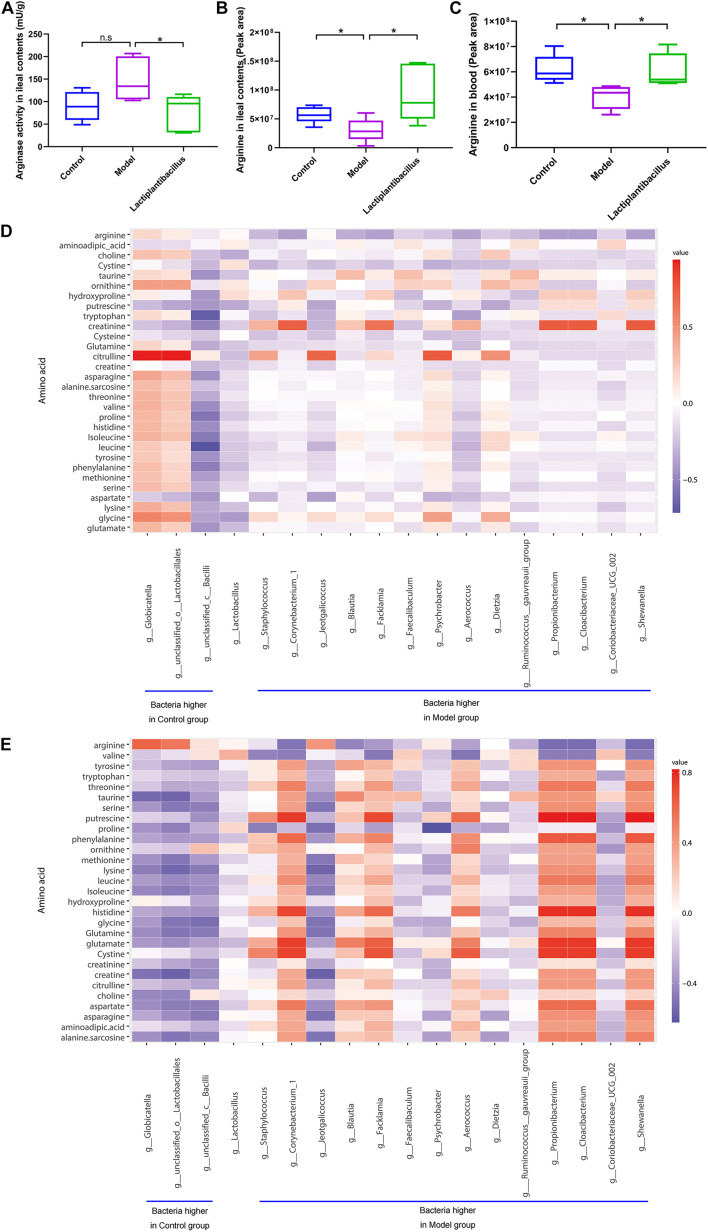
Arginase activity in ileal contents and arginine level in ileal contents and blood in the control group (*n* = 5), model group (*n* = 5), and *Lactiplantibacillus* group (*n* = 5). **(A)** The arginase activity in ileal contents. **(B)** The level of arginine in ileal contents. **(C)** The level of arginine in blood. **(D)** The correlation between the bacteria at higher abundance in the control group or model group in the ileum and amino acids in the ileal contents. **(E)** The correlation between the bacteria at higher abundance in the control group or model group in the ileum and amino acids in the blood. *, *P* < 0.05; n.s, *P* > 0.05.

To verify whether arginase and its metabolic substrate were involved in renal crystal formation, we examined the activity of arginase and found that it was lower in the ileal contents in the N-1-supplemented group than in the model group (Figure 3A). We also examined the levels of different amino acids in the ileal contents (Supplementary Table 3) and blood (Supplementary Table 4). The level of arginine in the ileal contents was higher in the control group (*P* = 0.040) and N-1-supplemented group (*P* = 0.029) than in the model group (Figure 3B). The blood arginine level was also lower in the model group (*P* = 0.010) than in the control and N-1 groups (*P* = 0.021) (Figure 3C). The ornithine and citrulline levels in the ileal contents and blood was not significantly different between three groups (Supplementary Figure 4). In addition, the level of arginine in the ileal contents (Figure 3D) or blood (Figure 3E) was positively correlated with the bacteria at greater abundance in the control group and was negatively correlated with the bacteria at greater abundance in the model group.

### Arginine Ameliorated Calcium Oxalate Crystal-Renal Cell Adhesion

**FIGURE 4:**
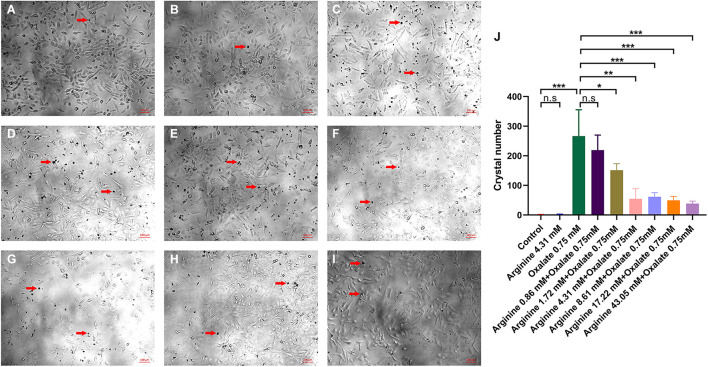
The effect of sodium oxalate and arginine on the adhesion of CaOx crystals to renal epithelial HK-2 cells. CaOx crystal adhesion to renal epithelial HK-2 cells after exposure to **(A)** normal saline, **(B)** 4.31 mM arginine, **(C)** 0.75 mM sodium oxalate, **(D)** 0.86 mM arginine + 0.75 mM sodium oxalate, **(E)** 1.72 mM arginine + 0.75 mM sodium oxalate, **(F)** 4.31 mM arginine + 0.75 mM sodium oxalate, **(G)** 8.61 mM arginine + 0.75 mM sodium oxalate, **(H)** 17.22 mM arginine + 0.75 mM sodium oxalate, **(I)** 43.05 mM arginine + 0.75 mM sodium oxalate (×100). The red arrow refers to the CaOx crystals. **(J)** Quantitative data for CaOx crystals adhering to the cells mentioned above. *, *P* < 0.05; **, *P* < 0.01; ***, *P* < 0.001; n.s, *P* > 0.05.

To explore whether arginine could affect crystal adhesion of renal epithelial cells *in vitro*, we added CaOx crystals and arginine to the cell culture medium. The results showed that there was no adhesion of CaOx crystals to renal epithelial HK-2 cells when cells were treated with was arginine alone. The crystals adhesion increased after the exposure to 0.75 mM oxalate, and reduced when cells were exposed to 0.75 mM oxalate + 1.72 mM or higher arginine (*P* = 0.023) (Figure 4).

## Discussion

Our previous study showed that *L. plantarum* N-1 was resistant to acid and bile salt and, compared to other strains, had the highest survival rates of 98.0 and 99.1% under the conditions of pH 3.5 and 0.1% bile salt, respectively. This strain has the ability to adhere to intestines and antibacterial activity. In addition, the culture supernatants of N-1 could inhibit the growth of *Escherichia coli* (Liu et al., 2017). All these features guaranteed the survival of *L. plantarum* N-1 in the intestinal tract.

In a normal healthy person, 2–10% oxalate in the food is absorbed into blood, while the remaining part is degraded by the gut microbiota. In addition, some dietary precursors (such as glyoxylate and hydroxyproline) are also metabolised to oxalate as a terminal metabolite production in the liver (Sadaf et al., 2017). The oxalate is excreted through kidney and the intestine. Thus, the gut microbiota may play a key role in regulating the oxalate metabolism. It was reported that *Lacticaseibacillus casei*, capable of degrading oxalate *in vitro*, reduced urinary oxalate excretion and renal crystals in rats (Kwak et al., 2006). However, this study did not explore the underlying mechanisms. Later, some studies found that *L. plantarum* recombined with the oxalate decarboxylase gene could also reduce urinary levels (Sasikumar et al., 2014; Paul et al., 2018). However, these artificial bacteria may not be safe for humans. Some clinical studies also found that probiotics containing *Lactobacillus* could decrease urinary oxalate levels in idiopathic hyperoxaluria or renal stone patients, but the decline was not significantly different between probiotic-treated patients and placebo-treated patients (Campieri et al., 2001; Lieske et al., 2005; Goldfarb et al., 2007; Siener et al., 2013). Interestingly, our study found that *L. plantarum* could reduce renal crystals without decreasing urinary oxalate excretion. Murphy et al. (2009) also reported that not all *Lactobacillus* could degrade oxalate. Thus, there may be some other mechanisms in addition to oxalate metabolism by which *L. plantarum* N-1 in our study reduced renal crystal deposition.

Several studies have reported that the gut microbiota is different between renal CaOx stone patients and healthy controls, which suggests that the whole gut microbiota, instead of a single kind of bacterium, is important in the formation of renal stones. Thus, we further examined the composition of the gut microbiota in the ileum, caecum, and colon between the three groups. We found that *Lactobacillus* was the predominant bacterium in the gut, especially in the ileum. We also found that the beta diversity of the gut microbiota and the percentage of *Lactobacillus* in the N-1-supplemented group were more similar to those in the control group, which indicated that N-1 could reverse the gut microbiota of model rats to relatively normal conditions. However, this phenomenon was not observed in the caecum or colon. The most likely reason may be that the environment in the ileum was more suitable for *Lactobacillus*.

We also found that *Lactobacillus* was the main genus of the gut microbiota. Its relative abundance was much higher in the colon of the control group, which suggested that the decrease in *Lactobacillus* was associated with renal stones. Our study also verified that supplementation with *L. plantarum* N-1 could reduce renal crystal formation. In addition, short-chain fatty acid (SCFA)-producing bacteria such as *Eubacterium* and *Ruminococcaceae* have been found in lower abundances in the gut microbiota of model groups (Wei and Tao, 2018; Xie et al., 2019). It was reported that SCFAs have anti-inflammatory functions (Koh et al., 2016). However, *Staphylococcus*, *Corynebacterium 1*, *Jeotgalicoccus*, *Psychrobacter*, and *Aerococcus* were higher in the gut microbiota of the model group of this study, and these genera are associated with acute or chronic inflammatory diseases, including inflammatory bowel diseases, necrotising lymphadenitis, and diabetes mellitus (El Mouzan et al., 2018; Salem et al., 2019; Xu et al., 2019; Zhou et al., 2019; Chen et al., 2020). This evidence suggested that N-1 could directly or indirectly ameliorate the dysbiosis of the gut microbiota and decrease inflammatory levels in various organs and tissues (such as kidneys) by regulating the gut microbiome, followed by less damage to renal cells and fewer crystals (Khan, 2014).

In this study, we predicted that a higher level of arginase would be found in the ileal microbiota of the model group than in the N-1-supplemented group. The predictive results also showed that the arginase level in caecal or colonic contents was not significantly different between model group and N-1-supplemented group. Meanwhile, the relative abundance of *Lactobacillus* was higher in the ileal contents than in the caecal and colonic contents. Thus, we hypothesised that the supplemented *Lactobacillus* mainly regulated the arginase activity and arginine level in the ileal contents. Further examinations verified that the arginase activity was lower in the ileal contents of N-1-supplemented rats than in the model rats. Arginine levels were higher in the ileal contents and blood of N-1-supplemented rats than in the model rats. Ornithine and citrulline are the key arginine metabolites, where arginine is metabolised to ornithine through arginase. Thus, we also compared the ornithine and citrulline levels in the ileal contents and blood. Though they were not significantly different between three groups, there was a decrease trend of ornithine level in the ileal contents after the supplementation of N-1.

Additionally, an *in vitro* study also showed that arginine could reduce CaOx crystal adhesion to renal cells. Kandhare et al. (2015) and Pragasam et al. (2005) reported that the administration of L-arginine could increase urinary citrate and reduce free radicals and oxalate in the kidney in rats with renal crystals. Kizivat et al. (2017) also found that L-arginine could reduce oxidative stress in kidney epithelial cells exposed to oxalate *in vitro*. Arginine could also suppress the inflammatory response and apoptosis of intestinal epithelial cells (Zhang et al., 2020a,b). All this evidence suggests that *L. plantarum* N-1 may reduce renal crystals by regulating the metabolism of gut microbiota and increasing the level of arginine in the gut.

The present study was performed only on Sprague–Dawley rats. The effect of N-1 on preventing renal CaOx stones in humans needs further research. However, our study also had several advantages. First, N-1, isolated from traditional cheese in China, is not pathogenic to humans and is easily obtained. Second, compared with the study performed by Kwak et al. (2006), our study is the first to explore the change in gut microbiota after the administration of *L. plantarum* and to discuss its effect on preventing renal CaOx stones by decreasing arginase levels in the gut microbiota. These results will promote further study of the mechanisms underlying renal CaOx stone formation.

## Conclusion

Our findings highlighted the important role of *L. plantarum* N-1 in reducing renal CaOx crystals by regulating arginine metabolism in the gut microbiota. Probiotics containing *L. plantarum* N-1 may be potential therapies for preventing renal CaOx stones.

## Data Availability Statement

The data presented in the study are deposited in the NCBI repository, accession number: PRJNA759335.

## Ethics Statement

The animal study was reviewed and approved by West China Hospital of Sichuan University Medical Research Ethics Committee.

## Author Contributions

YL, XJ, QS, and KW conceived and designed the experiments. YL, LT, YM, LC, and YC performed the experiments, analysed the data, and wrote the manuscript. ZJ, XJ, HL, QS, and KW reviewed and edited the manuscript. QS and KW provided guidance for the experiments. All authors contributed to the article and approved the submitted version.

## Conflict of Interest

The authors declare that the research was conducted in the absence of any commercial or financial relationships that could be construed as a potential conflict of interest.

## Publisher’s Note

All claims expressed in this article are solely those of the authors and do not necessarily represent those of their affiliated organizations, or those of the publisher, the editors and the reviewers. Any product that may be evaluated in this article, or claim that may be made by its manufacturer, is not guaranteed or endorsed by the publisher.
