# Supplementary material for: Lactiplantibacillus plantarum Reduced Renal Calcium Oxalate Stones by Regulating Arginine Metabolism in Gut Microbiota
Source: Front Microbiol. 2021 Sep 24;12:743097. doi: 10.3389/fmicb.2021.743097 (PMC8498331; doi:10.3389/fmicb.2021.743097)
Supplement: Supplementary file 1 [file Data_Sheet_1.DOCX]

Supplementary Material

**Supplementary Table 1.** The information of amino acids standards used for liquid chromatography-mass spectrometry analysis.

| **Amino acids standards** | **Source** | **Catalogue number** |
| --- | --- | --- |
| Creatine | Sigma | C0780 |
| L-Tryptophan | Sigma-Aldrich | T0254 |
| Creatinine | Sigma-Aldrich | C4255 |
| L-Citrulline | Sigma-Aldrich | C7629 |
| Sarcosine | Sigma-Aldrich | S7672 |
| L-Cystine | Sigma | 30200 |
| L-Histidine | Sigma-Aldrich | H8000 |
| L-Cysteine hydrochloride | Sigma | C1276 |
| L-Asparagine | Sigma | A0884 |
| Taurine | Sigma | T0625 |
| L-Ornithine monohydrochloride | Sigma-Aldrich | 57197 |
| DL-2-Aminoadipic acid | Sigma | A0637 |
| trans-4-Hydroxy-L-proline | Sigma-Aldrich | H54409 |
| Putrecine | Sigma-Aldrich | 51799 |
| L-Alanine | Sigma | YJ-140624 |
| Glycine | Sigma | YJ-140624 |
| D-Serine | Sigma | YJ-140624 |
| L-Proline | Sigma | YJ-140624 |
| D-Valine | Sigma | YJ-140624 |
| L-Threonine | Sigma | YJ-140624 |
| L-Isoleucine | Sigma | YJ-140624 |
| L-leucine | Sigma | YJ-140624 |
| D-Lysine | Sigma | YJ-140624 |
| Monosodium glutamate | Sigma | YJ-140624 |
| L-Glutamine | Sigma | YJ-140624 |
| DL-Methionine | Sigma | YJ-140624 |
| D-Arginine | Sigma | YJ-140624 |
| L-Tyrosine | Sigma | YJ-140624 |
| aspartate | Sigma | YJ-140624 |
| Choline | Sigma-Aldrich | A6625 |
| Phenylalanine | Sigma | 40541 |

**Supplementary Table 2.** Alpha diversity of the gut microbiota in the ileum, jejunum, caecum and colon between the control, model and *Lactiplantibacillus* groups.

| **Site** | **Alpha diversity indices** | **Control** | |  | **Model** | |  | **Lactiplantibacillus** | | ***P* value*** | ***P* value^†^** | ***P* value^‡^** |
| --- | --- | --- | --- | --- | --- | --- | --- | --- | --- | --- | --- | --- |
|  |  | **Mean** | **SD** |  | **Mean** | **SD** |  | **Mean** | **SD** |  |  |  |
| **Ileum** | Sobs | 77.80 | 28.28 |  | 106.10 | 45.46 |  | 87.70 | 54.03 | 0.12 | 0.21 | 0.94 |
|  | Ace | 145.21 | 66.98 |  | 215.77 | 123.90 |  | 169.57 | 115.05 | 0.21 | 0.31 | 0.91 |
|  | Chao | 112.09 | 44.71 |  | 168.01 | 69.37 |  | 139.32 | 85.63 | 0.08 | 0.31 | 0.62 |
|  | Shannon | 2.05 | 0.45 |  | 1.98 | 0.38 |  | 1.79 | 0.34 | 0.47 | 0.19 | 0.12 |
|  | Simpson | 0.24 | 0.11 |  | 0.25 | 0.08 |  | 0.28 | 0.07 | 0.43 | 0.62 | 0.43 |
|  | Coverage | 1.00 | 0.00 |  | 1.00 | 0.00 |  | 1.00 | 0.00 | 0.07 | 0.33 | 0.47 |
| **Cecum** | Sobs | 409.70 | 63.92 |  | 430.20 | 52.17 |  | 397.20 | 26.88 | 0.65 | 0.09 | 0.12 |
|  | Ace | 549.46 | 83.34 |  | 559.63 | 65.58 |  | 527.91 | 45.67 | 0.79 | 0.21 | 0.08 |
|  | Chao | 549.25 | 91.61 |  | 570.52 | 64.82 |  | 526.75 | 38.18 | 0.91 | 0.05 | 0.09 |
|  | Shannon | 3.99 | 0.48 |  | 4.09 | 0.33 |  | 3.87 | 0.28 | 0.85 | 0.12 | 0.34 |
|  | Simpson | 0.06 | 0.03 |  | 0.05 | 0.02 |  | 0.07 | 0.04 | 0.91 | 0.16 | 0.31 |
|  | Coverage | 0.99 | 0.00 |  | 0.99 | 0.00 |  | 0.99 | 0.00 | 0.94 | 0.24 | 0.20 |
| **Colon** | Sobs | 376.50 | 81.37 |  | 423.90 | 61.20 |  | 412.00 | 34.12 | 0.14 | 0.43 | 0.08 |
|  | Ace | 526.59 | 108.98 |  | 564.34 | 83.68 |  | 562.11 | 56.74 | 0.31 | 0.79 | 0.21 |
|  | Chao | 509.74 | 107.06 |  | 553.33 | 83.30 |  | 569.90 | 56.10 | 0.38 | 0.91 | 0.12 |
|  | Shannon | 3.55 | 0.51 |  | 4.05 | 0.32 |  | 3.99 | 0.29 | 0.01^a^ | 0.73 | 0.01^a^ |
|  | Simpson | 0.09 | 0.04 |  | 0.05 | 0.02 |  | 0.06 | 0.03 | 0.01^a^ | 0.57 | 0.04^a^ |
|  | Coverage | 0.99 | 0.00 |  | 0.99 | 0.00 |  | 0.99 | 0.00 | 0.45 | 0.91 | 0.29 |

SD, standard deviation.

*, *P* value between control and model groups, †, *P* value between model and Lactiplantibacillus groups, ‡, *P* value between control and Lactiplantibacillus groups.

a, *P* < 0.05.

**Supplementary Table 3.** The levels of different amino acids in the ileal contents of rats in the control, model and *Lactiplantibacillus* groups.

| **Amino acids** | **Control** | |  | **Model** | |  | **Lactiplantibacillus** | | ***P* value*** | ***P* value^†^** | ***P* value^‡^** |
| --- | --- | --- | --- | --- | --- | --- | --- | --- | --- | --- | --- |
|  | **mean** | **SD** |  | **mean** | **SD** |  | **mean** | **SD** |  |  |  |
| alanine/sarcosine | 3815968.90 | 2323200.79 |  | 2915554.85 | 460258.83 |  | 5766809.26 | 4225725.28 | 0.42 | 0.17 | 0.39 |
| aminoadipic acid | 1571.64 | 297.15 |  | 1568.88 | 236.91 |  | 1357.01 | 227.48 | 0.99 | 0.19 | 0.24 |
| arginine | 57738311.18 | 14354664.78 |  | 30537034.49 | 20349880.45 |  | 94082625.18 | 49256443.60 | 0.04^a^ | 0.03^a^ | 0.15 |
| asparagine | 814505.51 | 685184.57 |  | 709590.72 | 201206.78 |  | 1141082.78 | 546915.85 | 0.75 | 0.14 | 0.43 |
| aspartate | 438645.42 | 155731.55 |  | 635877.54 | 21078.69 |  | 1067539.21 | 615462.03 | 0.02^a^ | 0.16 | 0.06 |
| choline | 10199286.02 | 12000042.95 |  | 10887228.18 | 5120943.10 |  | 21236733.74 | 21083013.23 | 0.91 | 0.32 | 0.34 |
| citrulline | 4538475.05 | 4793525.78 |  | 1488146.67 | 819755.09 |  | 1813385.12 | 557325.34 | 0.20 | 0.48 | 0.24 |
| creatine | 1229988.91 | 1130950.56 |  | 1707698.57 | 607368.95 |  | 5900652.87 | 5887894.06 | 0.43 | 0.15 | 0.12 |
| creatinine | 47795188.20 | 32104670.61 |  | 73463497.54 | 51790796.71 |  | 56252228.71 | 17704949.03 | 0.37 | 0.50 | 0.62 |
| Cysteine | 1419.98 | 501.11 |  | 4485.74 | 3313.78 |  | 27994.31 | 33672.97 | 0.08 | 0.16 | 0.12 |
| Cystine | 15339.90 | 19611.58 |  | 8445.97 | 9351.35 |  | 41749.20 | 42183.16 | 0.50 | 0.12 | 0.24 |
| glutamate | 5390948.97 | 2902109.19 |  | 5019420.23 | 612564.34 |  | 6822071.08 | 3580839.48 | 0.79 | 0.30 | 0.51 |
| Glutamine | 3337117.16 | 3227206.91 |  | 3225163.39 | 1432772.18 |  | 7918219.73 | 5929891.08 | 0.95 | 0.12 | 0.17 |
| glycine | 255463.45 | 195094.36 |  | 239337.89 | 91768.11 |  | 250780.02 | 117809.68 | 0.87 | 0.87 | 0.96 |
| histidine | 16859702.91 | 12244816.32 |  | 14386303.41 | 4270530.14 |  | 23644493.56 | 11616659.26 | 0.68 | 0.13 | 0.39 |
| hydroxyproline | 102560.56 | 62256.85 |  | 82411.13 | 37818.96 |  | 72725.92 | 25828.79 | 0.55 | 0.65 | 0.35 |
| Isoleucine | 144388647.42 | 62024727.84 |  | 147626172.08 | 23060144.01 |  | 164293480.76 | 34788388.81 | 0.92 | 0.40 | 0.55 |
| leucine | 160477286.28 | 63396473.60 |  | 170092833.28 | 12044884.35 |  | 189927334.94 | 24048306.26 | 0.75 | 0.14 | 0.36 |
| lysine | 13045013.77 | 10191553.09 |  | 9524605.47 | 1357307.62 |  | 20553501.21 | 12671004.32 | 0.47 | 0.09 | 0.33 |
| methionine | 41372209.66 | 23346938.92 |  | 38450688.77 | 6131951.20 |  | 59956638.41 | 29122889.53 | 0.79 | 0.14 | 0.30 |
| ornithine | 1548426.79 | 831804.17 |  | 948791.92 | 1019557.61 |  | 590638.09 | 394597.49 | 0.34 | 0.48 | 0.05 |
| phenylalanine | 36648496.18 | 21465365.38 |  | 36852594.02 | 1874210.16 |  | 53979972.31 | 19491387.12 | 0.98 | 0.09 | 0.22 |
| proline | 67519219.47 | 40459391.92 |  | 68527912.43 | 10096908.62 |  | 87036891.48 | 27696386.44 | 0.96 | 0.20 | 0.40 |
| putrescine | 1335482.79 | 1046996.69 |  | 1367320.92 | 583344.27 |  | 2099192.44 | 1478575.39 | 0.95 | 0.33 | 0.37 |
| serine | 2049884.57 | 1681599.34 |  | 1586747.60 | 523897.61 |  | 3583871.62 | 2536876.90 | 0.57 | 0.12 | 0.29 |
| taurine | 1120256.06 | 749401.04 |  | 1288348.69 | 599965.66 |  | 1473096.02 | 882000.31 | 0.71 | 0.71 | 0.51 |
| threonine | 2999924.01 | 2231685.76 |  | 2250759.81 | 599027.72 |  | 4329727.43 | 2979565.70 | 0.49 | 0.16 | 0.45 |
| tryptophan | 27865076.58 | 17665064.04 |  | 31142007.94 | 8846826.52 |  | 40222685.91 | 9575105.24 | 0.72 | 0.16 | 0.21 |
| tyrosine | 37343103.22 | 24843335.87 |  | 37261438.09 | 3010597.24 |  | 61771002.69 | 30631133.01 | 0.99 | 0.11 | 0.20 |
| valine | 71625532.49 | 44570413.09 |  | 66420244.69 | 14304523.37 |  | 91519664.80 | 37337028.99 | 0.81 | 0.20 | 0.47 |

SD, standard deviation.

*, *P* value between control and model groups, †, *P* value between model and Lactiplantibacillus groups, ‡, *P* value between control and Lactiplantibacillus groups.

a, *P* < 0.05.

**Supplementary Table 4.** The levels of different amino acids in the blood of rats in the control, model and *Lactiplantibacillus* groups.

| **Amino acids** | **Control** | |  | **Model** | |  | **Lactiplantibacillus** | | ***P* value*** | ***P* value^†^** | ***P* value^‡^** |
| --- | --- | --- | --- | --- | --- | --- | --- | --- | --- | --- | --- |
|  | **mean** | **SD** |  | **mean** | **SD** |  | **mean** | **SD** |  |  |  |
| alanine/sarcosine | 18407654.20 | 5177255.65 |  | 20332484.02 | 5577926.80 |  | 22123344.39 | 5799706.48 | 0.59 | 0.63 | 0.32 |
| aminoadipic acid | 173848.09 | 60052.75 |  | 203970.57 | 106464.82 |  | 192135.02 | 126591.35 | 0.60 | 0.88 | 0.78 |
| arginine | 61962362.13 | 11190565.89 |  | 40130081.71 | 9428333.05 |  | 61084041.83 | 13392480.53 | 0.01^a^ | 0.02^a^ | 0.91 |
| asparagine | 3109164.51 | 1365408.60 |  | 3396740.50 | 1152302.88 |  | 3784619.92 | 1307432.88 | 0.73 | 0.63 | 0.45 |
| aspartate | 552410.18 | 399177.76 |  | 1197847.90 | 566042.33 |  | 1143829.67 | 563699.76 | 0.07 | 0.88 | 0.09 |
| choline | 23019018.90 | 5811917.43 |  | 29635251.86 | 6349301.08 |  | 25428586.68 | 4283345.65 | 0.12 | 0.25 | 0.48 |
| citrulline | 18724711.55 | 5331680.50 |  | 20718861.47 | 8811931.33 |  | 20975456.48 | 8259387.04 | 0.68 | 0.96 | 0.62 |
| creatine | 29647926.35 | 7600158.83 |  | 40017120.84 | 6719578.66 |  | 38028124.99 | 10765431.53 | 0.05 | 0.74 | 0.19 |
| creatinine | 37417115.00 | 6708590.39 |  | 39322612.49 | 6778496.58 |  | 39887029.01 | 4457858.70 | 0.67 | 0.88 | 0.51 |
| Cystine | 607206.07 | 286362.51 |  | 701471.18 | 562011.06 |  | 671363.86 | 264728.93 | 0.75 | 0.92 | 0.72 |
| glutamate | 14128266.89 | 5485607.65 |  | 30226204.41 | 13523503.54 |  | 20514123.05 | 9467532.94 | 0.04^a^ | 0.22 | 0.23 |
| Glutamine | 70083177.94 | 12777271.23 |  | 81040461.91 | 15607968.75 |  | 83094996.37 | 12978041.74 | 0.26 | 0.83 | 0.15 |
| glycine | 1144379.07 | 463694.05 |  | 1425403.17 | 323561.06 |  | 1528896.50 | 394465.50 | 0.30 | 0.66 | 0.20 |
| histidine | 29610462.49 | 12197505.92 |  | 39624551.65 | 23528366.94 |  | 35184294.93 | 11105695.26 | 0.42 | 0.71 | 0.47 |
| hydroxyproline | 5711946.73 | 1654312.13 |  | 5272093.61 | 1264453.15 |  | 5591450.83 | 2115146.51 | 0.65 | 0.78 | 0.92 |
| Isoleucine | 55118538.82 | 11589664.22 |  | 63498245.87 | 16225355.95 |  | 63734385.74 | 14054849.42 | 0.37 | 0.98 | 0.32 |
| leucine | 71461001.87 | 12739621.26 |  | 82703274.55 | 23316854.51 |  | 81745821.21 | 16911451.03 | 0.37 | 0.94 | 0.31 |
| lysine | 76310734.63 | 16635907.29 |  | 88811975.90 | 17412933.54 |  | 92494213.69 | 13091308.94 | 0.28 | 0.72 | 0.13 |
| methionine | 10360702.98 | 2175823.73 |  | 12029828.56 | 1695502.73 |  | 11990158.48 | 3162950.24 | 0.21 | 0.98 | 0.37 |
| ornithine | 15931691.75 | 7700334.79 |  | 14733498.48 | 7215479.41 |  | 14790631.03 | 7135304.90 | 0.81 | 0.99 | 0.81 |
| phenylalanine | 17722643.41 | 5246397.94 |  | 24503495.59 | 10915006.08 |  | 19857924.65 | 5880471.78 | 0.25 | 0.43 | 0.56 |
| proline | 14046168.45 | 3967412.81 |  | 12751027.14 | 2822278.94 |  | 15511006.39 | 2588267.40 | 0.57 | 0.15 | 0.51 |
| putrescine | 552063.12 | 260985.38 |  | 816494.01 | 662692.34 |  | 604464.97 | 219274.01 | 0.43 | 0.52 | 0.74 |
| serine | 10909186.45 | 3874912.83 |  | 12904473.38 | 3537892.04 |  | 13696779.28 | 4303566.52 | 0.42 | 0.76 | 0.31 |
| taurine | 393960.62 | 210103.70 |  | 751672.39 | 178794.67 |  | 596450.62 | 163416.98 | 0.02^a^ | 0.19 | 0.13 |
| threonine | 15303289.06 | 5469318.24 |  | 17524297.70 | 5843766.25 |  | 17033287.75 | 5522641.27 | 0.55 | 0.89 | 0.63 |
| tryptophan | 35221479.65 | 11360968.96 |  | 38663807.69 | 15529337.28 |  | 40168645.86 | 18872328.17 | 0.70 | 0.89 | 0.63 |
| tyrosine | 1383032.35 | 330094.82 |  | 1614895.06 | 337912.91 |  | 1494994.88 | 522452.34 | 0.30 | 0.68 | 0.70 |
| valine | 44478616.87 | 8805938.78 |  | 43758809.67 | 8356825.82 |  | 41117677.83 | 8606128.89 | 0.90 | 0.64 | 0.56 |

SD, standard deviation.

*, *P* value between control and model groups, †, *P* value between model and Lactiplantibacillus groups, ‡, *P* value between control and Lactiplantibacillus groups.

a, *P* < 0.05.


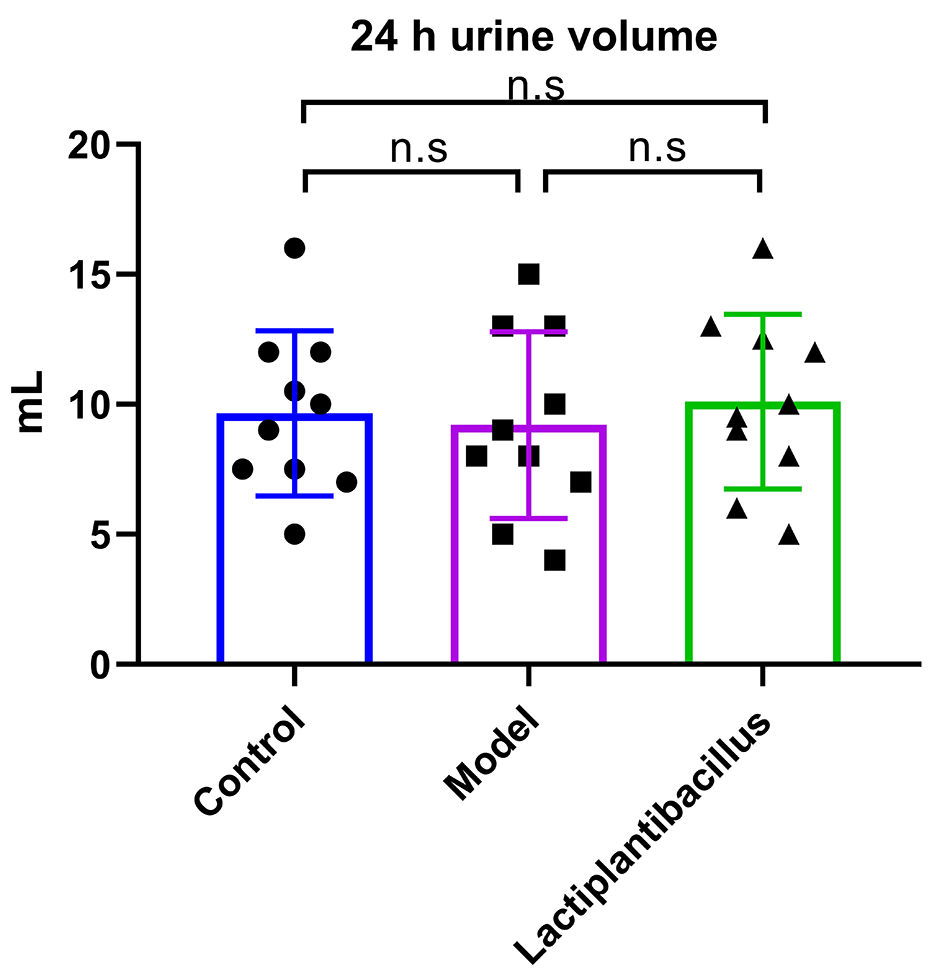


**Supplementary Figure 1.** The volume of 24 h urine of rats in the control group (n = 10), model group (n = 10) and Lactiplantibacillus group (n = 10). Data are presented as the mean ± standard deviation and were analyzed using Student’s t-test between two groups. n.s, *P* > 0.05.


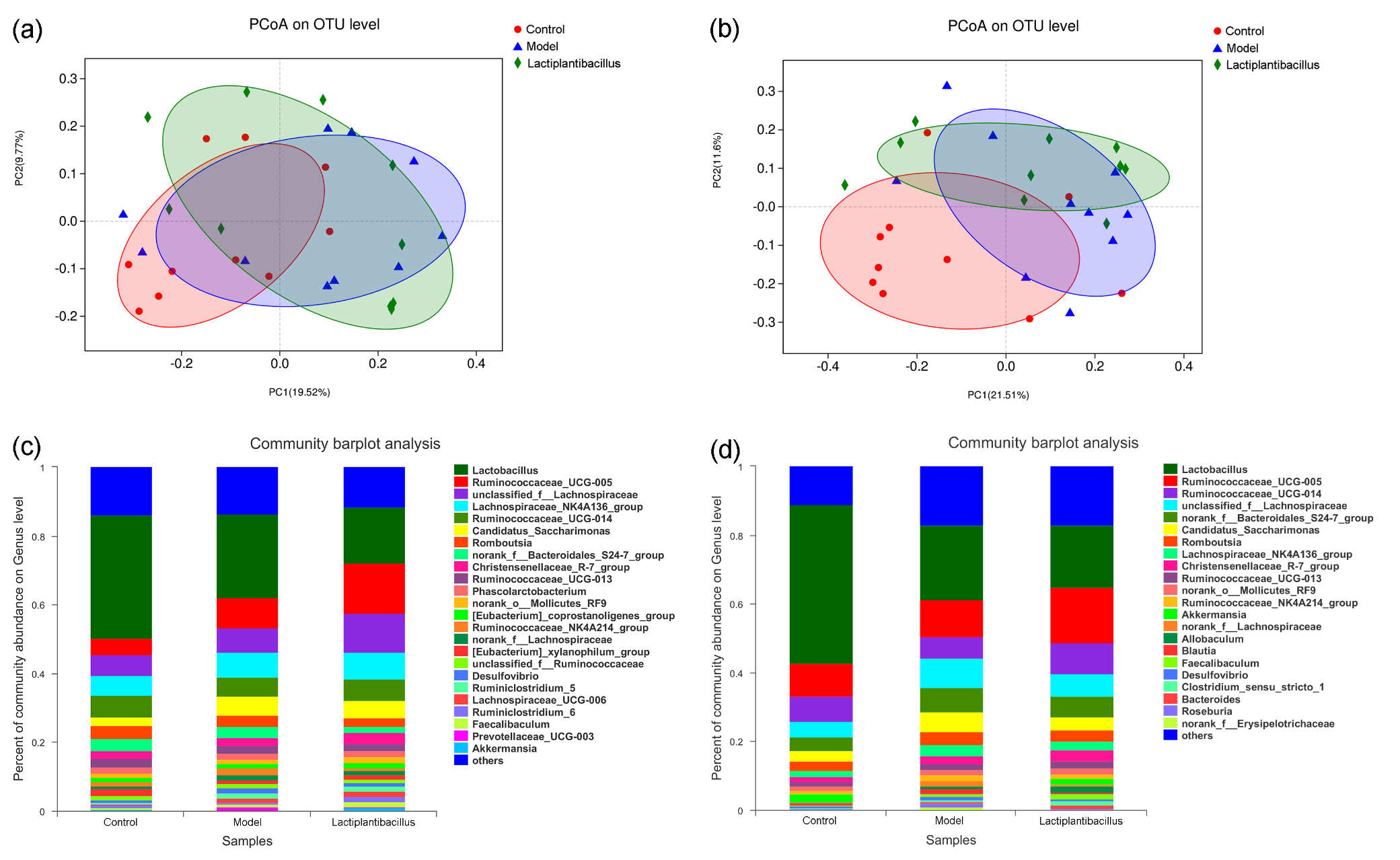


**Supplementary Figure 2.** Beta diversity and components of the gut microbiota in the caecum and colon between the control group (n = 10), model group (n = 10) and *Lactiplantibacillus* group (n = 10). Beta diversity of the gut microbiota in the (a) caecum and (b) colon between the three groups was assessed by principal coordinate analysis using the Bray-Curtis index. The relative abundance of bacteria at the genus level in the (c) caecum and (d) colon between the three groups is presented by the colourful columns


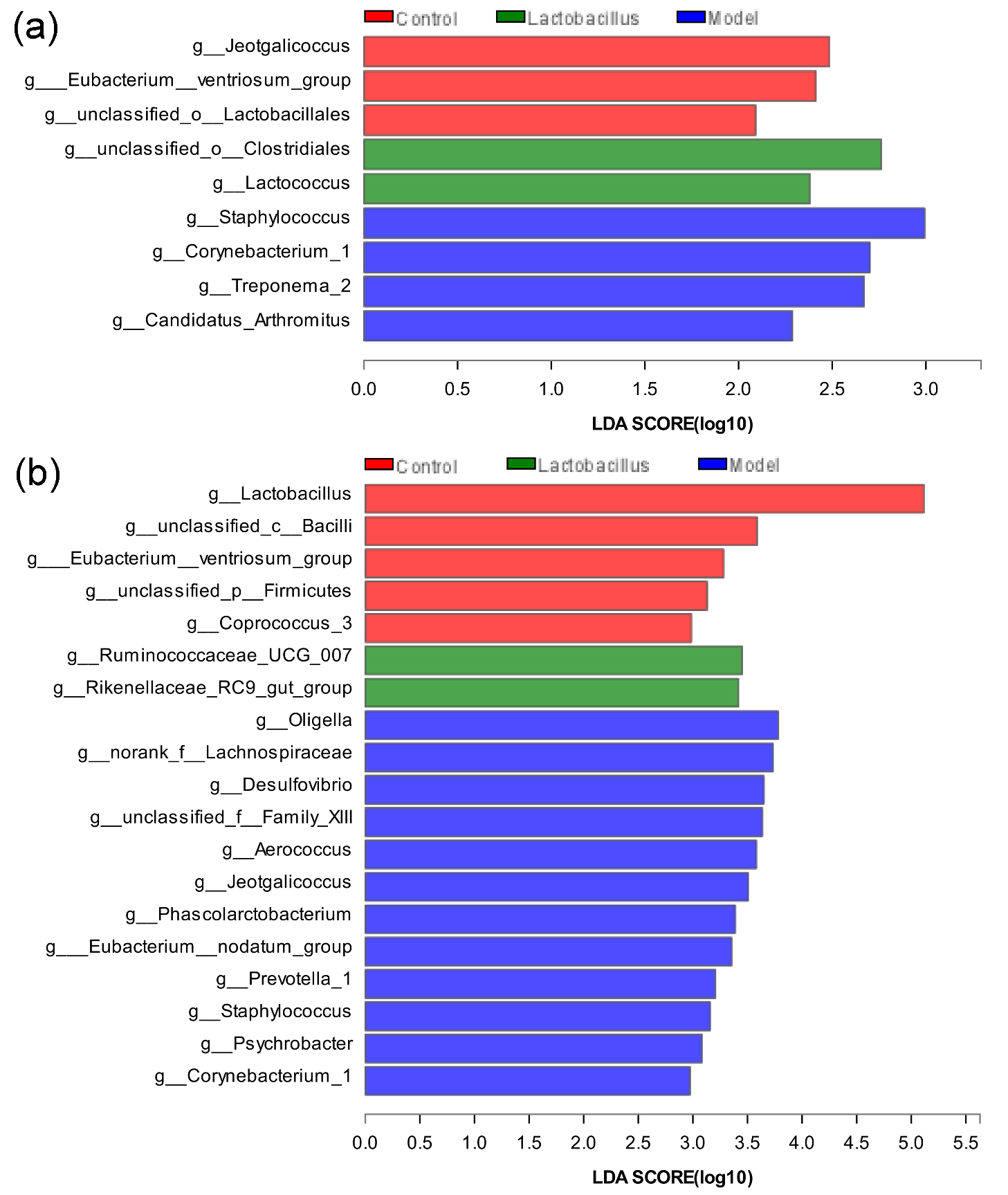


**Supplementary Figure 3.** Discriminative gut bacteria between the model group (n = 10) and *Lactiplantibacillus* group (n = 10). Plots show the bacteria with higher relative abundance in the control group (red bars), model group (blue bar) and *Lactiplantibacillus* group (green bars) using linear discriminant analysis effect size (LEfSe) at the genus level in the (a) caecum and (b) colon (*P* < 0.05).


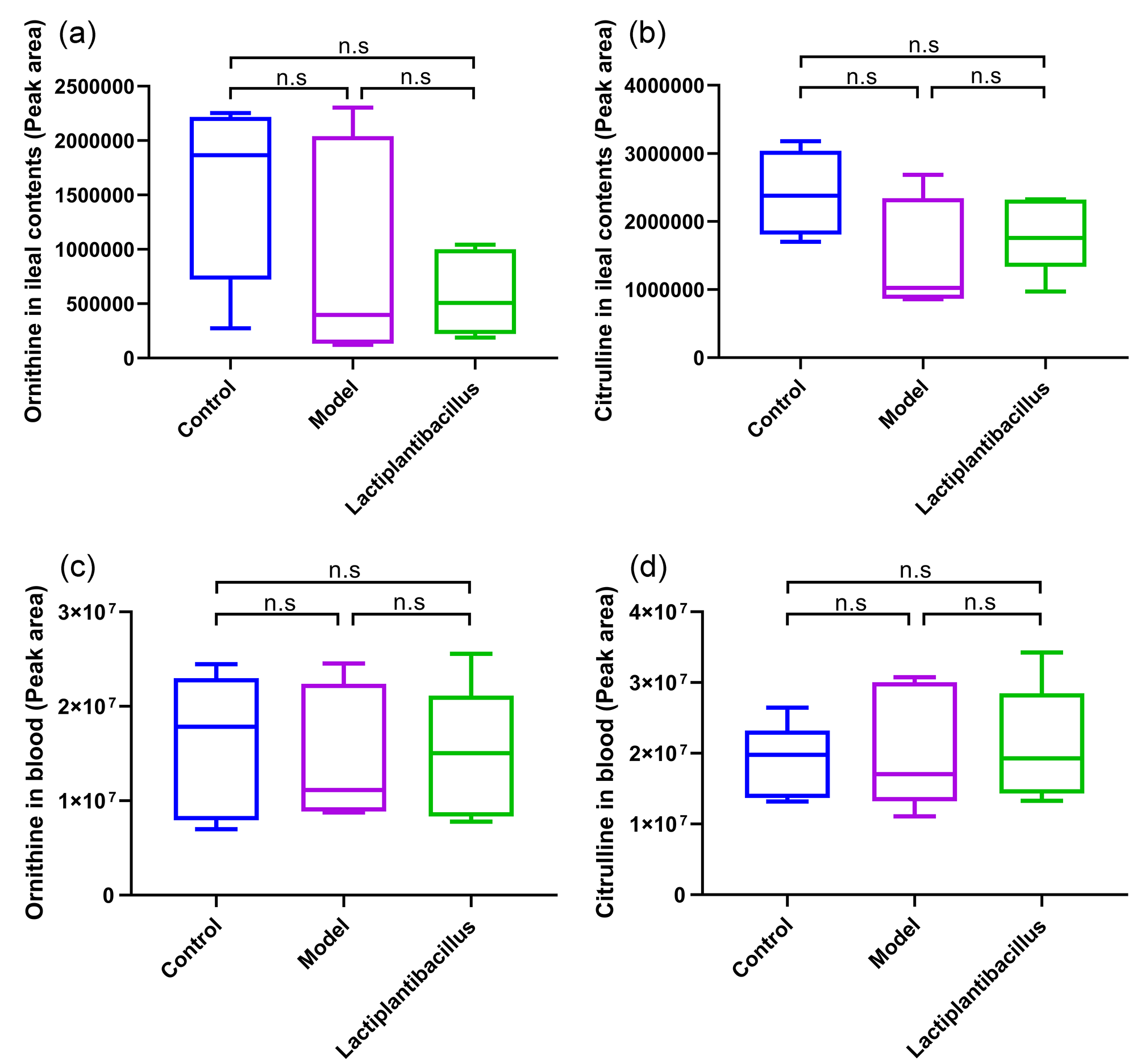


**Supplementary Figure 4.** The levels of ornithine and citrulline in the ileal contents and blood. (a) The level of ornithine in the ileal contents. (b). The level of citrulline in the ileal contents. (c) The level of ornithine in the blood. (d). The level of citrulline in the blood. Data are presented as the mean ± standard deviation and were analyzed using Student’s t-test between two groups. n.s, P > 0.05.
